# The Correlation between Stigma and Family Acceptance with Religiosity of PLWH MSM in Medan, Indonesia

**DOI:** 10.31372/20190403.1048

**Published:** 2019

**Authors:** I Nyoman Arya Maha Putra, Agung Waluyo, Sri Yona

**Affiliations:** a STIKES Bali, Denpasar, Bali, Indonesia; b Department of Medical Surgical Nursing, Faculty of Nursing, Universitas Indonesia, Depok, West Java, Indonesia

**Keywords:** stigma, family acceptance, religiosity

## Abstract

There are several factors related to religiosity of PLWH MSM in Medan. This study aimed to identify the correlation between stigma and family acceptance with religiosity of PLWH MSM in Medan. This cross-sectional study applied purposive sampling technique and involved 175 samples from H. Adam Malik Public Central Hospital, Medan Pirngadi Hospital, Padang Bulan and Teladan Public Health Centers. The data was analyzed using the multiple logistic regression technique. Bivariate analysis showed a significant correlation between stigma along with family acceptance with religiosity of PLWH MSM in Medan (*p* = 0.005; *α* = 0.005).

## Introduction

Human Immunodeficiency Virus (HIV) is a major global health issue infecting more than 35 million people. Approximately, 1 million people died of Acquired Immunodeficiency Syndrome (AIDS) worldwide in 2016 (WHO, 2018). In Indonesia, the Ministry of Health’s Progress Report on HIV-AIDS in 2016 showed an increase in HIV cases from 30,935 in 2015 to 41,250 in 2016 (Kemenkes, 2016). North Sumatra is in the top 10 provinces in Indonesia with an increase in new HIV cases from 1,491 in 2015 to 1,891 in 2016 (Dinas, 2015).

A group at risk for HIV/AIDS is men who have sex with men (MSM).^1^ The Joint United Nations Programme on HIV and AIDS (UNAIDS) data reported an increase of people living with HIV (PLWH) MSM in China from 1.5% in 2005 to 8.0% in 2015 (Duan et al., 2017). Based on Indonesia’s Ministry of Health data (2016), 4,241 Men Having Sex with Men (MSM) have suffered HIV and the number has increased to 13,063 in 2016 (Kemenkes, 2017). Data by North Sumatra’s Board of Health showed an increase percentage of PLWH MSM from 4% in 2012 to 10% in 2015 (Dinas, 2015).

A prevalent increase of HIV cases in MSM has shown HIV transmission enhancement, especially through risky sexual behavior (Li et al., 2017; Maria et al., 2017; Pan et al., 2015; Seyedalinaghi et al., 2016; Shang & Li, 2013), defined as sexual behavior that leads to HIV transmission risk (Black & Hawks, 2014). Risky sexual behavior can occur due to several factors, one of which is religiosity.

Religion is a belief and practice of approaching or communicating to God (Koenig, 2012; Reed & Neville, 2014; The American Academy of HIV Medicine, 2017). Religiosity in PLWH MSM tends to increase after HIV is diagnosed. Previous research has suggested that religiosity is associated with a variety of HIV high-risk behaviors and one study stated that high religiosity is associated with low risky sexual behavior (Shaw, Saifi, Lim, & Saifuddeen, 2017; Szaflarski, 2013; Watkins, Simpson, Cofield, Davies, & Usdan, 2015).

When PLWH MSM realize the importance of religion’s role in their lives, risky sexual behavior can be avoided. Religions constrain their believers to not engage in any risky behaviors, including risky sexual behavior (Garofalo et al., 2016; Ilesanmi & Alele, 2014; Shaw et al., 2017). In addition, religion can also be a mental health treatment for PLWH MSM. Thus, PLWH MSM becomes more involved in healthy behavioral efforts (Jeffries et al., 2014).

Religiosity is related with stigma. High stigma on PLWH MSM causes many negative impacts in daily lives, such as depression (Berg & Ross, 2017; Rueda et al., 2016; Smit, Brady, Carter, Fernandes, & Lamore, 2012). When in depression, the religiosity of PLWH MSM decrease. Previous studies suggested that when a person’s religiosity is high, his depression level would decrease. This is because when a person improves his relationship with God and others, he actually is distracted and has no time to descend deeper into the depression (Basri, Hong, & Oon, 2014; Othman et al., 2015).

In addition, family acceptance is also associated with religiosity. Family is smallest unit in the community. Parents in particular are a vital component for PLWH MSM (Li et al., 2009; Qypi, 2017; Ryan, 2009). The role of families is to provide positive support that can significantly affect mental health, especially to ease depression (Mitrani et al., 2017; Qypi, 2017; Ryan, 2009). Family acceptance opens a possibility for PLWH MSM to improve their behavior by sharing the religious values. We aimed at identifying whether stigma and family acceptance are correlated with religiosity in PLWH MSM in Medan.

## Methods

The purposive sampling method was applied in cross-sectional studies with 175 samples from H. Adam Malik Public Central Hospital, Medan Pirngadi Hospital, and Padang Bulan and Teladan Public Health Centers between January and June 2018. There was no characteristic differences among respondents in hospital and public health centers. Data were collected from April 24 to May 21, 2018 by researcher who had previously passed the ethical test in Faculty of Nursing, Universitas Indonesia with number 157/UN2/F12.D/HKP.02.04/2018.

Prospective respondents were MSM who came to take routine medicine in the hospital or public health centers. Identification of respondents was assisted by nurses who work in the hospital or public health centers and gained from medical records before they were given a screening test and had obtained permission previously from prospective respondent. The screening test was intended to ensure or confirm that prospective respondents were MSM. Inclusion criteria were: age range above 18 years, able to read and write in Bahasa Indonesia, have no mental disorder, and provide informed written consent to participate in the study. Exclusion criterion included whether the subject was hospitalized or being recommended for hospitalization.

When the prospective respondent met the criteria and willing to participate they were asked to sign the informed consent followed by filling out the questionnaire. The process of filling the questionnaire was approved by the respondents and performed in a specific room prepared by researchers and designed to maintain the respondents’ privacy. Participating respondents were given fees as gratitude. The respondent still have the right to refuse to participate in this study.

The research instruments used were the religious level questionnaire, Perceived Acceptance Scale (PAS), and Berger HIV Stigma Questionnaire. Religious level questionnaire and PAS were adjusted accordingly. Validity and reliability tests were performed on these instruments with correlation coefficient 0.365–0.820 and Cronbach’s alpha 0.931 and 0.913, respectively. Berger HIV Stigma Questionnaire was already in Indonesian form with correlation coefficient 0.98 and Cronbach’s alpha 0.94.

## Results

The demographic characteristics revealed that most respondents were young adults or at least 40 years old (93.7%) with a high school education level (95.4%) and 92% worked and had income less than the Provincial Minimum Salary (50.9%). Most were unmarried (90.6%) and 61.1% were diagnosed with HIV for about 18 months. Seventy-two respondents (41%) adhered to Muslim, 46 respondents (26%) adhered to Catholicism, 33 respondents (19%) adhered to Protestantism and 24 respondents (14%) adhered to Buddhism. There was no significant relationship among demographic characteristics with religiosity.

The result for stigma, family acceptance and religiosity are shown in Table 1 whilst relationship of stigma and family acceptance with religiosity are shown in Table 2. Table 1 shows that most respondents obtained good family acceptance (53.7%), but that the stigma obtained was high (51.4%). Religiosity of respondents was mostly high (52%). Table 2 revealed that family acceptance and stigma were related to religiosity (*p* <  0.001, *p* = 0.005 respectively with *α* = 0.05). Family acceptance had a positive correlation with religiosity, whereas stigma had negative correlation. These results showed that good family acceptance and low stigma are associated with high religiosity.

**Table 1:** Stigma, Family Acceptance, and Religiosity among PLWH MSM in Medan

Characteristic	Total
	(*N* = 175)
Family acceptance	
Good	94 (53.7%)
Poor	81 (46.3%)
Stigma	
High	90 (51.4%)
Low	85 (48.6%)
Religiosity	
Good	91 (52%)
Poor	84 (48%)

**Table 2:** Relationship between Stigma and Family Acceptance with Religiosity among PLWH MSM in Medan

	Religiosity	OR (95% Confidence Interval)
	(*X*^2^)	
Family acceptance	0.000^*^	7.402 (3.787–14.467)
Poor		
Good		
Stigma	0.005^*^	2.495 (1.357–4.589)
High		
Low		

Multivariate analysis showed that family acceptance was related to religiosity, while stigma became a confounding variable. According to the odds ratio (OR) score, good family acceptance will increase religiosity by up to seven times more than poor family acceptance after controlling for stigma.

## Discussion

Religion has an important role in the lives of PLWH MSM. Religion as a belief and practice is a mean of communication with God and has a positive impact on PLWH MSM. One of which is to avoid high-risk behaviors of HIV transmission, especially risky sexual behavior. This finding is similar to result of the previous findings that explained that the role of religion reduces risky sexual behaviors through religious environment and teachings (Garofalo et al., 2016; Mojahed, 2014; Shaw et al., 2017). Religion can be a treatment in terms of PLWH MSM mental health. When these men make mistakes and feel that they deserved to be infected with HIV, religion denies this feeling and provides motivation to increase the subject’s self-worth. Therefore, PLWH MSM can be increasingly involved in healthy behavioral efforts (Jeffries et al., 2014).

The analysis showed that religiosity of respondents was high with good family acceptance. However, the stigma of PLWH was high. Religiosity of respondents in this study was related to family acceptance and stigma of PLWH MSM. According to the OR score, good family acceptance will increase religiosity by up to seven times more than acceptance of poor families after controlling for stigma.

Good family acceptance makes it possible for PLWH MSMs to set their religiosity to the high level. This is because through family acceptance they obtained meaningful support. Family support for PLWH MSM can be diversed from financial support, daily activities, medication, and psychological support (Li et al., 2009; Mitrani et al., 2017; Ryan, 2009).

Psychological problems often appear in PLWH MSM, one of which can be depression caused by high stigma (Berg & Ross, 2017; Rueda et al., 2016; Smit et al., 2012). Through family support, depression levels of PLWH MSM will decline. With decreasing depression level, families can improve the religious teachings of PLWH MSM to increase their religiosity. Therefore, religiosity may keep PLWH MSM distracted so that they do not descend deeper into depression, can be more productive, and can avoid risky sexual behaviors (Basri et al., 2014; Ryan, 2009).

The limitations of this study include: (1) the study was limited to Medan city so that the results described only one area and (2) initial planning of this research used simple random sampling technique. However, due to limited time to implement it, the researcher then used the purposive sampling technique. Therefore, the result cannot be generalized to the target population. However, this study also has advantages that the adequate sample of 175 respondents was quite representative of the affordable population.

## Conclusion

Religiosity was correlated with family acceptance. Good family acceptance can increase religiosity by up to seven times higher than poor family acceptance after controlling for stigma. Good family acceptance provides positive support for PLWH MSM to avoid negative impacts, especially depression which leads to risky sexual behaviors. With decreasing depression levels, families can improve the religiosity of PLWH MSM through religious teachings.

## Recommendation

If family acceptance is promoted and negative stigma is diminished through health services, mainly nursing, religiosity can have an optimum role in decreasing risky sexual behaviors.

## Declaration of Conflicting Interests

The authors declared no potential conflicts of interest with respect to the research, authorship, and/or publication of this article.

## Funding

This work is supported by Hibah PITTA 2018 funded by DRPM Universitas Indonesia No. 1829/UN2.R3.1/HKP.05.00/2018. The research process has received a lot of support. The appreciations go to the respondents who have been willing to involve and the Directors of H. Adam Malik Public Central Hospital and Medan Pirngadi Hospitals and Heads of Puskesmas Padang Bulan and Puskesmas Teladan.
